# Through-polymer, via technology-enabled, flexible, lightweight, and integrated devices for implantable neural probes

**DOI:** 10.1038/s41378-024-00691-8

**Published:** 2024-04-22

**Authors:** Cunkai Zhou, Ye Tian, Gen Li, Yifei Ye, Lusha Gao, Jiazhi Li, Ziwei Liu, Haoyang Su, Yunxiao Lu, Meng Li, Zhitao Zhou, Xiaoling Wei, Lunming Qin, Tiger H. Tao, Liuyang Sun

**Affiliations:** 1 College of Electronics and Information Engineering, Shanghai University of Electric Power, Shanghai, China; 2 2020 X-Lab, Shanghai Institute of Microsystem and Information Technology, Chinese Academy of Sciences, Shanghai, China; 3 School of Graduate Study, University of Chinese Academy of Sciences, Beijing, China; 4 State Key Laboratory of Transducer Technology, Shanghai Institute of Microsystem and Information Technology, Chinese Academy of Sciences, Shanghai, China; 5 Center of Materials Science and Optoelectronics Engineering, University of Chinese Academy of Sciences, Beijing, China; 6 School of Physical Science and Technology, ShanghaiTech University, Shanghai, China; 7 Center for Excellence in Brain Science and Intelligence Technology, Chinese Academy of Sciences, Shanghai, China; 8 Neuroxess Co., Ltd. (Jiangxi), Nanchang, Jiangxi China; 9 Guangdong Institute of Intelligence Science and Technology, Hengqin, Zhuhai, Guangdong China; 10 Tianqiao and Chrissy Chen Institute for Translational Research, Shanghai, China

**Keywords:** Engineering, Biosensors

## Abstract

In implantable electrophysiological recording systems, the headstage typically comprises neural probes that interface with brain tissue and integrated circuit chips for signal processing. While advancements in MEMS and CMOS technology have significantly improved these components, their interconnection still relies on conventional printed circuit boards and sophisticated adapters. This conventional approach adds considerable weight and volume to the package, especially for high channel count systems. To address this issue, we developed a through-polymer via (TPV) method inspired by the through-silicon via (TSV) technique in advanced three-dimensional packaging. This innovation enables the vertical integration of flexible probes, amplifier chips, and PCBs, realizing a flexible, lightweight, and integrated device (FLID). The total weight of the FLIDis only 25% that of its conventional counterparts relying on adapters, which significantly increased the activity levels of animals wearing the FLIDs to nearly match the levels of control animals without implants. Furthermore, by incorporating a platinum-iridium alloy as the top layer material for electrical contact, the FLID realizes exceptional electrical performance, enabling in vivo measurements of both local field potentials and individual neuron action potentials. These findings showcase the potential of FLIDs in scaling up implantable neural recording systems and mark a significant advancement in the field of neurotechnology.

## Introduction

Implantable electrophysiological recording systems are cornerstone technologies in neuroscience, playing a pivotal role in advancing our understanding, diagnosis, and treatment of neural disorders^1–3^. These systems are critical for in vivo extracellular recording and offer unparalleled insights into neural activity. A recording system typically comprises three main components: implantable neural probes that interface directly with brain tissue^4–6^, integrated circuit (IC) chips for signal refinement^7^, and a host computer that executes system control and data analysis. Because the majority of accessible neurons produce signals marginally above the noise floor (approximately 5–20 μV)^8,9^, integrating amplifiers on the chip itself represents a compelling approach^10–12^. This integration, which is ideally on the same substrate as the electrode array, is proposed to diminish load capacitance and mitigate signal attenuation, which is essential for preserving the fidelity of the biological signals captured^13–16^.

Along with the advancement of signal amplification techniques, the field has made concerted efforts to enhance brain activity mapping by expanding the number of recording channels on both implantable probes and IC chips^17–19^. This scale-up is driven by the desire for more comprehensive neuronal recordings. However, a corresponding challenge has been to minimize the physical footprint of these components, thereby reducing their influence on the natural behavior of research subjects wearing recording devices^20–22^. Tracing the technological evolution since the 1950s reveals a remarkable transition from metal microwires and bulky transistors to the sophisticated realms of microelectromechanical systems (MEMS) and microelectronics^17,23–25^, which have significantly augmented overall recording capacities. Exemplary innovations include the Michigan probe and Utah array^26–28^, which utilize MEMS technology, and state-of-the-art complementary metal oxide semiconductor (CMOS) systems^29–31^, such as the Argo system, which supports 65,536 recording channels^32^.

Despite significant advancements in MEMS and microelectronics, the integration of these implantable components with external systems remains a critical challenge^33–35^. Traditional methods, such as using gold wire bundles and percutaneous connectors, add considerable weight and have proven to be reliability concerns. It is impractical to fan out channels and create adapters when channel counts reach the thousands. Although Neuropixel offers an integrated probe and application-specific integrated circuit (ASIC)^36^, its high cost and limited customizability underscore the need for innovation. The emergence of flexible neural probes, which improved integration with brain tissue, exacerbates connection challenges due to their inherent flexibility.

The international technology roadmap for semiconductors identifies the integration of MEMS with ICs as a crucial ‘More-Than-Moore’ technology^37^. This is commonly addressed through a multichip approach, combining separately fabricated MEMS and IC components into a system-in-package or vertical multichip module, known as three-dimensional integrated circuits (3D ICs). Inspired by the through-silicon via (TSV) method from 3D IC techniques^37^, this work introduces the innovative through-polymer via (TPV) method, which involves crafting a flexible, lightweight, integrated device (FLID) that overcomes the need for traditional adapters. This vertical stacking method pairs a homemade flexible probe with a commercial Intan amplifier chip (Intan Technologies) directly onto a printed circuit board (PCB) compared with conventional headstages on which the area and weight are reduced by more than 50 and 75%, respectively. The FLID significantly reduced the torque applied on the animal head to ~0.03 N·mm, less than one-eighth that of conventional devices, facilitating greater freedom of movement and quicker postimplantation recovery; these behaviors are nearly the same as those of unimplanted control animals.

The FLID was rigorously tested for its electrical, mechanical, and biological properties. Surface modification with a platinum-iridium (Pt-Ir) alloy^38,39^ reduced the impedance of the gold recording sites to as low as approximately 60 kΩ at 1 kHz, enhancing the signal acquisition capability without requiring extra electroplating, as is often required for flexible probes. Mechanical testing confirmed that our flexible probes have an ultimate tensile strength of approximately 0.1 N, ensuring safe removal postexperiment while minimizing the risk of brain trauma. Immunohistochemical analysis at two months postimplantation in mice demonstrated the device’s durability and minimal tissue damage. Together with our custom main control software, the FLID captured high-quality neural signals in healthy and epileptic mouse models^40,41^, recording a spike amplitude of ~200 μV with an average signal-to-noise ratio (SNR) of 12.7 postsort. These results establish FLID as a transformative tool for electrophysiological recording, offering significant improvements over existing systems.

## Results

### The design and fabrication of FLIDs

To advance neuroscientific research, the engineering of next-generation brain-computer interfaces (BCIs) for use in animal studies is pivotal. Traditional neural implants, while effective, often impose significant physical constraints on research subjects due to their bulk and rigidity, thus influencing natural behaviors and affecting the validity of experimental data. To mitigate these limitations and enhance the welfare of animal subjects, our team prioritized the development of an FLID. This device represents a paradigm shift toward minimally invasive and highly adaptable neural recording systems, enabling more accurate and ethically responsible research.

**Fig. 1:**
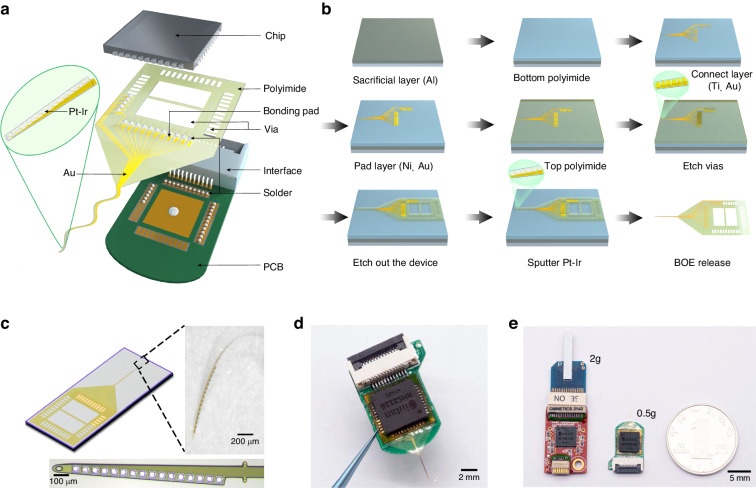
Design and fabrication of the FLID. **a** Exploded three-dimensional schematic illustrating the component layers and assembly of the FLID. **b** Sequential fabrication process flowchart for the flexible neural probe. **c** Fabricated flexible probe; magnified image of the probe immersed in phosphate-buffered saline. The bottom is a detailed view of the recording site at the probe’s front-end. **d** Assembled FLID held with tweezers, showing the compact integration of the device. **e** Comparative visualization of the size and weight between the SP and the FLID, highlighting the significant reduction achieved with the FLID design

The FLID integrates a chip, a flexible probe, and a PCB using TPV, realizing a compact sandwich structure (Fig. 1a). This innovative design establishes a direct connection between the flexible probe and the chip, considerably reducing the volume and weight of the resulting device. The small footprint and lightness of the FLID are critical for reducing encumbrance in animal subjects, thereby preserving their natural behavior during neural signal acquisition and daily activities.

In our design, we employed the TPV method, which is sufficiently versatile to be applied to most packaged chips, including quad flat no-leads package (QFN) and ball grid array (BGA) chips. For this study, we selected the QFN packaged Intan chip (model number RHS2116), a widely used amplifier in the neuroscience field, to demonstrate our method. Our flexible probe is bifurcated into two primary sections: the back end, which connects to the external electrical system, and the front end, which is implanted into brain tissue.

To ensure geometric compatibility with the Intan chip, the back end of the flexible probe was fabricated to align with the chip’s readout channels. Specifically, the chip features 44 peripheral pins and 1 central pin (Supplementary Fig. 1), with 16 pins designated for neural signal acquisition and 29 pins for communication with the host computer software. Correspondingly, the back end of our flexible probe was designed with 16 bonding pads and 29 VIAs (Supplementary Fig. 2). These bonding pads comprise nickel/gold electrodes exposed by etching and were connected to the chip’s signal acquisition pins. Moreover, the vias, which were etched through polyimide, facilitate a secure bond between the chip and the PCB.

At the front end of the system, to access neural signals in deeper brain regions, our probe features a single shank design. The shank, measuring 75 µm in width, is equipped with 16 recording sites evenly distributed along its length of 1 mm, with each site being 50 × 50 µm to provide high spatial resolution.

The device substrate, an ultrasmall PCB, was meticulously designed to complement the dimensions of both the chip and the flexible probe. The dimensions of the PCB are a mere 17 × 10 mm with a thickness of only 0.5 mm. At one extremity, it includes a flexible printed circuit (FPC) interface for external neural signal processing system connectivity. The PCB’s center houses a 44-pin gold socket, tailored for the QFN of the chip (Supplementary Fig. 3).

The fabrication of flexible probes is a testament for the meticulous application of MEMS technology (Fig. 1b), with a strong emphasis on biocompatibility and mechanical flexibility. We encapsulated gold wiring in a polyimide film approximately 4 µm thick, leveraging the proven MEMS process stability and biocompatibility of polyimide^42,43^. The multistep fabrication sequence includes metal deposition, polyimide solidification, gold evaporation, and another polyimide layer, followed by reactive ion etching (RIE) device etching to expose the recording sites, bonding pads, and vias. These sites are further coated with a platinum-iridium alloy to optimize signal acquisition. Subsequently, the probes are released from the wafer using a Buffered Oxide Etch (BOE) solution. Importantly, we observe that the final probe is completely freestanding and flexible without any silicon substrate, in contrast to previously reported flexible devices^34^^,^ which usually use a silicon wafer as the back end for connecting to an external PCB, which contributes to minimizing the total weight of the headstage.

As depicted in Fig. 1c, the manufactured probe demonstrates outstanding flexibility, retaining its structural integrity when curled within phosphate-buffered saline (PBS). Optical microscopy further confirmed the consistent and uniform distribution of the recording sites. We used double-sided polyimide tape to affix the flexible probe onto the PCB substrate, followed by the application of solder across all pads and vias. The Intan chip, which amplifies, filters, and digitizes the neural signals, is securely bonded atop the probe using reflow soldering with a durable connection (Fig. 1d) (see the detailed bonding process in the Methods section). Moreover, compared with conventional devices, FLIDs present a substantial reduction in both size and weight, with reductions of more than half in area and 75% in weight (Fig. 1e).

### Evaluation of locomotor behavior in mice using open-field tests

In the preceding section, we established that FLIDs were significantly lighter than conventional FLIDs. Here, we next systematically demonstrated that a lighter design allows mice to become more active than a conventional bulky headstage. The open field test is a standard behavioral experiment used to evaluate locomotor activity and exploratory behavior in rodents, providing insight into their functional state. A primary challenge in such experiments is ensuring that implantable devices used for recording neural activity do not impede the natural movements of mice. To assess the impact of our FLID on the unrestricted movement of mice, we conducted a comparative study. We compared the locomotor activity of three groups of mice, with three mice in each group. The first group was implanted with FLID, and the second group was implanted with a conventional silicon probe (SP) in the same brain regions. A control group of unimplanted Blank mice served as the benchmark for uninhibited activity.

**Fig. 2:**
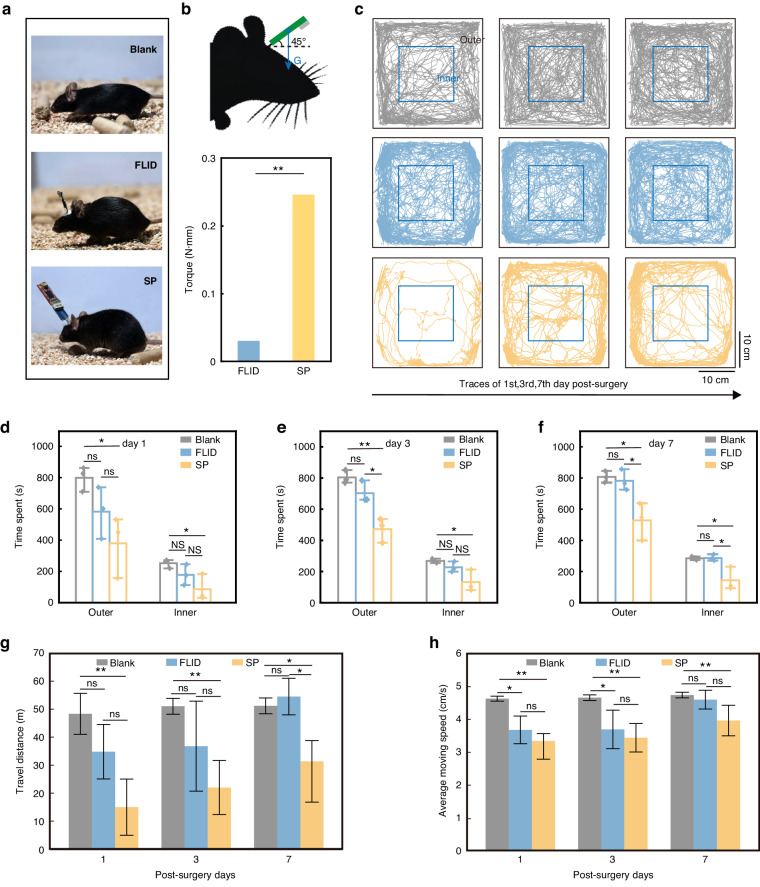
Evaluation of locomotor behavior in mice using open-field tests. **a** Images depicting three groups of mice engaged in activity: Blank, FLID and SP. **b** Graph showing the torque applied to the mice’s heads by the implanted devices. **c** Comparative travel traces of blank (gray), FLID (blue), and SP (yellow) mice over 30 min on Days 1, 3, and 7 postsurgery, illustrating the differences in locomotor activity. Statistical analysis of the time spent by the Blank, FLID, and SP mice in the outer and inner zones of the testing arena on Days 1 (**d**), 3 (**e**), and 7 (**f**) postsurgery. **g** Graph comparing the total travel distance of mice across the three groups over the course on Days 1, 3, and 7 postsurgery. **h** Graph illustrating the average movement speeds of blank, FLID, and SP mice on Days 1, 3, and 7 postsurgery. For all groups, *n* = 3 mice. Statistical significance was determined by t tests, denoted as ns (not significant, *p* ≥ 0.05), (*p < 0.05), and (**p < 0.01)

The devices were affixed to the heads of the mice using dental cement to ensure that they remained secure during active movements. We quantified the mechanical impact of the devices by analyzing the torque exerted on the heads of the mice raised to a 45° angle from the horizontal plane. The model revealed that the FLID exerted a torque of only 0.029 N·mm, which was significantly less than the 0.245 N·mm exerted by the SP (Fig. 2b). This disparity signifies a substantial enhancement in movement freedom facilitated by the FLID.

To assess the impact of our device on postsurgical activity, we monitored the mice in a new environment and captured their movement trajectories. Activity was analyzed from 30-min video segments recorded on days 1, 3, and 7 postsurgery. The movement patterns of the blank (control), FLID, and SP groups are represented by gray, blue, and yellow traces, respectively (Fig. 2c). These traces indicated a general increase in activity levels over time across all groups. Notably, the FLID group’s movement patterns closely mirrored those of the blank group, while the SP group showed a marked deviation.

In particular, we observed distinct differences in how the mice explored the outer and inner areas of the enclosure. On the first day postsurgery, FLID mice demonstrated a more extensive range of movement, utilizing almost the entire cage area. This finding starkly contrasted that of the SP mice, which exhibited a more limited range of motion. Quantitatively, FLID mice traveled 34.86 ± 9.7 m, which was significantly greater than the 15.06 ± 10 m traveled by SP mice (Fig. 2g). Furthermore, in terms of time spent in different areas of the enclosure, FLID mice were more active in both the outer (582 ± 165 s) and inner zones (177 ± 67 s) than were SP mice, who spent only 379 ± 197 s in the outer areas and a mere 86 ± 85 s in the inner areas (Fig. 2d). These data suggest that FLID mice maintain greater exploratory behavior and mobility. By the seventh day postsurgery, both FLID and SP mice showed increases in their overall activity. However, the activity levels of the FLID mice closely matched those of the Blank mice, with a significant reduction in time spent in the outer area, indicating a return to normal exploratory behavior^44^. Specifically, the FLID group had a travel distance of 54.53 ± 6.51 m, compared to 28.12 ± 11.16 m for the SP group (Fig. 2g). The times spent on the outer and inner activities were 782 ± 68 s and 287 ± 21 s, respectively, for FLID mice, whereas they were 528 ± 120 s and 144 ± 76 s, respectively, for SP mice (Fig. 2f). These findings highlight the effectiveness of FLID in supporting natural movement and exploration postsurgery.

In addition to total travel distance analyses, we also evaluated the average moving speeds of the mice, calculated from the effective distance traveled and the time spent. Seven days postsurgery, the speeds recorded for the Blank (control), FLID, and SP mice were 4.74 cm/s, 4.6 cm/s, and 3.97 cm/s, respectively (Fig. 2h). Notably, while the SP mice demonstrated only 80% of the speed of the control group after a week of recovery, the FLID mice demonstrated speed that closely approximated that of the control mice, indicating that implantation had a minimal impact on the FLID mice. This near-complete recovery in mobility among the FLID mice, compared to the slower movement in the SP group, underscores the effectiveness of FLID in preserving natural behaviors postsurgery.

We note that the above calculations of torque and behavioral experiments did not account for the influence of tethering wires on mouse activity during in vivo recordings. The impact of the cable on animal behavior can vary significantly and is influenced by factors such as the type of commutator used, the cable weight balancing system, and the position of the mice in the arena.

However, even when considering these cable-related factors, animals equipped with the FLID demonstrated increased activity in the open-field arena compared to those using the commercial Intan headstage. This enhanced activity is partly attributable to our selection of a lightweight cable (JS05B-12P-1000-4-8) for the FLID, in contrast to the more substantial RHS Stim SPI interface cable used with the Intan system. As depicted in supporting materials Supplementary Fig. 11a, a 1.8 m long JS05B-12P cable weighs approximately 4 g, which is significantly less than the 22.33 g weight of an equivalent length of the SPI interface cable.

To substantiate this observation, we present exemplary trajectory data from two animals during a 10-min recording session, each equipped with FLID and Intan ’system cables and the opposite end suspended 0.5 m above the center of the arena (Supplementary Fig. 11b). Notably, both subjects had their probes implanted for 3 months. The trajectory analysis showed that mice with the Intan system were more inclined to stay near the corners of the arena, whereas the FLID-equipped mice exhibited more foraging behavior, consistent with the findings shown in Fig. 2c.

The quantitative results for the behavioral parameters strongly support the notion that reducing the volume and weight of BCI devices substantially benefits the free activity of mice. Lessening head load may alleviate anxiety, enhance security, and improve the overall activity level of mice, thereby offering a more accurate representation of their behavior for analysis. The FLID design significantly improved the balance between sophisticated neural recording capabilities and ethical considerations of animal welfare.

### Electrical, mechanical, and biological characterization of FLID

We next systematically characterized the electrical, mechanical and biological properties of our FLID device. The impedance of the flexible probe is a critical determinant of neural signal quality. In our design, the chip is directly bonded to the back end of the probe, where the metal seeding layer is only a few hundred nanometers thick^45^. This naturally prompts an inquiry into the ability of the back end to maintain robust ohmic contact with the chip. Moreover, the reduced line width of the conductive traces in our ultraflexible probe design increases the impedance compared to that of traditional metal microwire probes. To enhance the electrical performance, we added extra nickel/gold layers, which served as the seeding layers on the back end to facilitate TPV bonding, while utilizing Pt-Ir alloy as the surface material for the front end electrical contacts, modified from previous recipe (detailed in the Methods section).

Scanning electron microscopy (SEM) inspection confirmed that the recording sites were uniformly coated with Pt-Ir. To quantify the electrical impedance, we employed an electrochemical workstation and obtained a mean impedance of 83.4 kΩ at 1 kHz from 18 recording sites across three different probes, which conforms to standards of the neuroscience community for action potential recording and is even lower than that of previously reported flexible probes that underwent poly(3,4-ethylene dioxythiophene) (PEDOT) electroplating^46,47^. The achievement of such low impedance not only indicates successful bonding at the back end but also establishes the device’s capability for electrical recording, which will be further discussed in subsequent sections.

We conducted both in vitro and in vivo tests to evaluate the long-term stability of the flexible Pt-Ir probe. In the in vitro experiment, the probe was immersed in PBS heated to 60 °C to accelerate the aging process and closely mimic physiological conditions (Supplementary Fig. 8a). Impedance measurements from 50 recording sites were taken after 1, 2, and 3 weeks of immersion in heated PBS, corresponding to equivalent durations of 5, 10, and 15 weeks at body temperature. These measurements were recorded as 241 kΩ, 305 kΩ, and 296 kΩ at 1 kHz, respectively. The results indicated no significant degradation in the stability of Pt-Ir over this period (Supplementary Fig. 8b). We further confirmed the stability by in vivo experiments. First, we measured the impedance of the electrodes implanted in three mice once a week for 4 weeks. The data, presented in Supplementary Fig. 8c, indicate that the impedance values remained stable throughout this duration. An essential aspect of our investigation into the mechanical properties of FLID was determining the robustness of its fully flexible probe, particularly its ability to be safely and entirely removed postexperimentation. To address this, we performed a mechanical strength test on the shank of the probe. The probe was secured on a testing bench, and a mechanical sensor simulated the action of extracting the probe from brain tissue. This test revealed that the shank withstood forces up to approximately 0.1 N before fracturing. On the other hand, to evaluate the force required to extract the probe from the brain, we follow the theoretic model^48^ by assuming that the probe is a cylindrical structure with a diameter of 100 µm; however, this model overstates the actual size of the probe, and the required extraction force is approximately 0.8 × 10^−3^ N, which is still far below the fracturing force of the probe.

We then conducted removal surgery on mice that had been implanted with the FLID for 2 months. The procedure was successful, as the probe was completely removed from the brain tissue without any damage to the probe or adverse effects on the health of the mice (Supplementary Fig. 4). The mice exhibited normal behavior following explant surgery, reinforcing the mechanical suitability of the FLID for in vivo studies. In addition, we were able to recycle the Intan chip postexperiment, which significantly lowered the cost of the FLID (see the detailed recycling process in the Methods section).

**Fig. 3:**
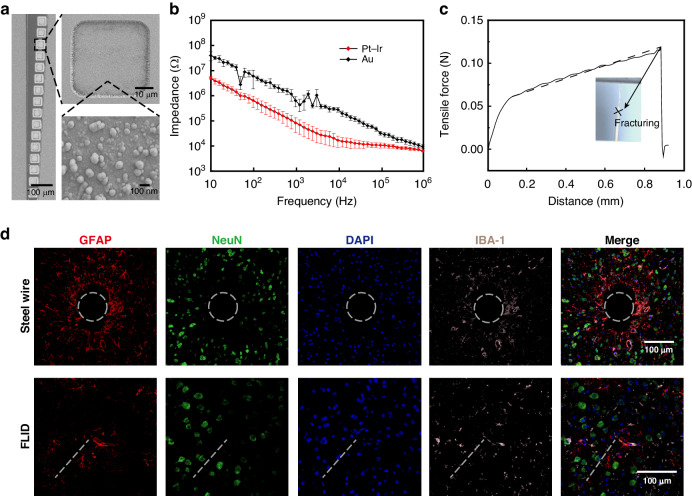
Characterization of the electrical, mechanical and biological properties of FLIDs. **a** Scanning electron microscope image depicting the Pt-Ir film on the surface of the recording site. **b** Impedance spectrum of 18 recording sites across 3 devices in PBS, comparing unmodified recording sites (Au) with those modified recording sites by Pt-Ir across a range of frequencies. **c** Graph illustrating the tensile force applied to the flexible probe shank during the fracturing process, with the inset showing an illustration of the fracture. **d** Immunohistochemical analysis of brain tissue 2 months postimplantation, contrasting the responses to a standard steel wire versus the FLID. Confocal fluorescence images display astrocyte activity (GFAP, red), neuron presence (NeuN, green), nuclei (DAPI, blue), and microglial response (IBA-1, pink), with merged views highlighting the differences between the steel wire and FLID implant sites

In addition to its mechanical robustness, the ultrathin and flexible design of the FLID probe offers exceptional biocompatibility, which is a crucial factor for long-term implantation. To further assess the biocompatibility, we conducted a comparative study of the FLID and a 100 µm diameter stainless steel wire, each of which was implanted into the mouse brain. After two months, immunohistochemical analysis was employed to evaluate the tissue response. At the site of the implanted stainless steel wire, a notable accumulation of astrocytes was observed without any surviving neurons. In contrast, the dotted position indicates the implantation site of the flexible probe, where there is a scarcity of astrocytes and living neurons (Fig. 3d). The results indicated that in the vicinity of the FLID, there was less glial scar formation and a lower incidence of neuronal death than in areas surrounding the stainless steel wire. This suggests that the implantable FLID component not only meets the mechanical demands of safe removal but also supports favorable biological integration over extended periods.

### Neural signal acquisition capability of FLID

**Fig. 4:**
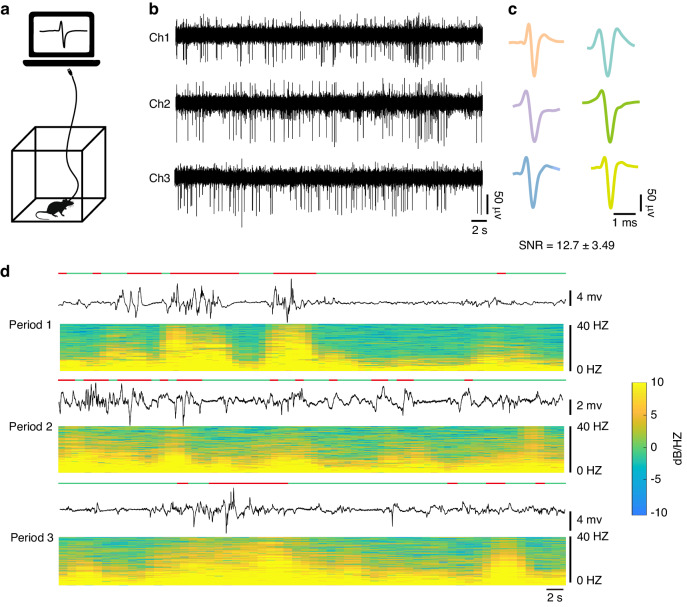
Acquisition and Analysis of Neural Signals in healthy and epileptic Mice. **a** Schematic representation of the neural signal acquisition setup with a mouse in an open-field environment. **b** Raw neural signal traces from three channels of a freely moving healthy mouse recorded one week postimplantation and filtered with a highpass filter at 300 Hz, demonstrating 20 s of data capture. **c** Extracted spikes from the raw signals using Mountainsort 5 with custom parameters, showing the identified neuronal activity and the calculated SNR of 12.7 ± 3.49. **d** One-minute segments of neural signals from three channels during various stages of seizures in an epileptic mouse, accompanied by corresponding low-frequency spectrograms to illustrate changes in neural dynamics across different periods. The red line delineates the periods where the mean amplitude of neural signals—computed over a one-second window—surpasses the 500 μV threshold, indicative of heightened seizure activity. Conversely, the green line identifies intervals where the neural signal amplitude remains below 500 μV, suggesting reduced seizure activity

While the ex vivo tests established the basic performance metric of our device, the true measure of success for the invasive BCI lies in the quality of the neural signals captured in vivo. To evaluate this capability, we implanted FLID into the hippocampus of both healthy and epileptic mice. Following a week-long recovery period, the mice were placed in an environment allowing free movement. A custom field programmable gate array (FPGA) device and our host computer software (Supplementary Fig. 5) were connected to the FLID via an FPC to facilitate interaction (Fig. 4a).

For the healthy mice, we filtered the acquired data between 300–6000 Hz. Based on the data from three representative channels at the same time (Fig. 4b), we were able to sort out six neurons with amplitudes up to ~200 μV, indicating substantial neural data collection capability. The spike waveforms are shown in Fig. 4c, with an average SNR of 12.7. This highlights the FLID’s ability to accurately record the activity of multiple neurons. We verified the stability of long-term in vivo recordings by collecting data for four weeks. Neural signals recorded with the flexible probe during this period showed no notable decrease in the SNR, as evidenced in Supplementary Fig. 8e. Specifically, we successfully recorded neural action potentials with a flexible probe up to four weeks postsurgery. For the 33 neurons recorded in total, the SNRs were 11, 10.9, 11.7, and 12.4 over the four-week period. This consistency demonstrates the reliable recording capabilities of the FLID.

In the epileptic mouse model, epilepsy was induced using a penicillin dosage of 7 million U/kg. Approximately 20 min postadministration, the mice exhibited symptoms such as limb twitching and abnormal crawling behaviors. During this period, we recorded and analyzed neural signals, confirming that the observed signals were genuine neural activity rather than motion-induced artifacts. FLID effectively captured neuronal activity during seizures. As depicted in Fig. 4d, a 1-min waveform window recording period different from that of the epileptic mice showed signal amplitude variations that correlated with behavioral changes. Strong twitching episodes in the mice coincided with amplitudes ranging from −5.2 mV to 6.3 mV^49,50^. Notably, our recording demonstrated that pronounced twitching episodes were typically associated with periods where the mean signal amplitude—computed over a 1-s window—exceeds 500 μV; these instances are indicated by the red line in the timeline, in contrast to the green line, which denotes lower amplitude periods. This observation reinforces FLID’s ability to precisely represent abnormal neuronal discharge activity, further confirming its exceptional neural signal acquisition proficiency in both healthy and pathological states.

## Discussion and conclusion

In summary, the adoption of the TPV method has allowed us to create a flexible and lightweight FLID for invasive BCIs. The diminutive and pliable nature of FLID is well suited to a variety of small animal models, setting it apart from traditional flexible electrodes. Its small size, light weight, and high degree of integration effectively mitigate issues such as the hindrance of mouse movement due to the bulky and heavy nature of existing devices, which can impact the animals’ daily activities. Notably, our FLID reduces the total weight of a 16-channel headstage to just 0.5 g, which is merely 25% of that of its conventional counterparts, resulting in a more than twofold increase in travel distance postimplantation.

Enhanced electrical properties are achieved by surface-modifying the electrode contacts with a platinum-iridium alloy, which resulted in an impressively low impedance of hundreds of kilohm at 1 kHz, enabling high-quality recordings of both local field potentials and single-neuron action potentials. This significant improvement in impedance facilitates virtually unrestricted movement in mice. The direct integration of the flexible probe and chip onto a PCB minimizes the presence of additional interfaces, streamlining neural signal collection. Moreover, the ’FLID design allows for nondestructive removal and potential reuse after experiments without causing secondary harm to the mice, offering a novel solution for small animal behavioral analysis.

While this study utilized a commercial 16-channel Intan chip for demonstration purposes, the TPV design principle is scalable to other high-channel-count amplifier chips and customized flexible probes. In conventional systems, adapters such as those in the Spikegadgets system (SpikeGadgets)—supporting up to 1024 channels—contribute significantly to the volume and weight, where FLID’s methodology is expected to reduce the total weight.

Despite its numerous advantages, the FLID also presents opportunities for future enhancements. Progressing toward ultrahigh integration, future iterations should strive to eliminate wired connections in favor of integrated wireless technologies such as WiFi or Bluetooth and utilize ultrathin polymers for device substrates. Additionally, incorporating stimulation capabilities within the probes could provide therapeutic interventions alongside flexible recording. This progress will pave the way for even more versatile BCIs that combine diagnostic and therapeutic functionalities.

## Methods

### Fabrication of fully flexible neural probes

We used standard MEMS processing technology to fabricate flexible probes. First, we used sputtering technology to make 1000 nm thick Al as a sacrificial layer, spin-coated 2 µm thick PI on the wafer, and solidified it in a 350 °C nitrogen oven for 8 h as the bottom insulation layer. Then, we fabricated recording sites and interconnects by lithography and electron beam evaporation, including a 5 nm thick Ti layer, a 100 nm thick Au layer, and a 5 nm thick Ti layer. Bonding pads were fabricated using a similar process, including a 5 nm thick Ti layer, a 150 nm thick Ni layer, and a 50 nm thick Au layer. Another layer of 2 µm thick PI is solidified on the top as an insulating encapsulation layer. A 100 nm thick layer of Al was sputtered on the wafer surface as a hard mask protection, and we performed RIE to expose the recording sites and bonding pads. Then, a 150 nm thick layer of Ni and a 50 nm thick layer of Au were again evaporated on the bonding pads, and a 3 nm thick layer of Cr and a 100 nm thick layer of Pt-Ir were sputtered at the recording site. Finally, after the wafer underwent dicing into individual dies, each piece was immersed in BOE solution for approximately 4 h at room temperature. The probes were then carefully retrieved with plastic tweezers, rinsed thoroughly in deionized water to remove any residual BOE solution, and dried on clean, dust-free paper. The released flexible probes are subsequently stored in a gel box for protection until use.

In particular, the etching of polyimide vias is indeed a pivotal step, and we employed the RIE method for this purpose. The fabrication process, as depicted in supporting materials Supplementary Fig. 6, involves the following steps:

1. An aluminum (Al) mask with a thickness of 100 nm was sputtered onto the top polyimide layer (approximately 2 µm thick).
2. The Al mask was patterned through photolithography and subsequent aluminum corrosion processes to define the areas for etching. First, the LC100A photoresist was spin-coated on an Al mask with a thickness of 2.1 µm, an exposure time of 7 s, and a development time of 60 s in a positive developer. Then, the aluminum etching solution was used to remove the Al mask in the etching area, and the temperature was approximately 20 s at 45 °C. Finally, the wafer was cleaned with deionized water.
3. The first RIE phase is conducted to etch the top polyimide layer, exposing the recording sites and bonding pads. Pure oxygen acts as an etching agent, with a flow rate of 45 sccm and an applied power of 500 W. This setup achieved an etch rate of approximately 0.3 µm per minute, and the etching time was approximately 7 min. To mitigate potential overheating, we employed multiple short etching sessions, each lasting between 30 and 60 s of active etching. The completion of the etching process was verified using a step profile measurement to ensure that the polyimide was fully cleared.
4. Remove the Al mask. Repeat step (2) to remove the Al mask by using an aluminum etching solution.
5. The second RIE phase is achieved by repeating steps (1–4) to etch through the dual layers of polyimide, shaping the flexible ’probe contour and forming the polyimide vias. The etching thickness of this step is approximately 4 µm, and the etching time is approximately 15 min.

### Bonding of FLID

To assemble the FLID, we first positioned the fabricated PCB substrate under a stereomicroscope. Laser-processed double-sided polyimide tape was then carefully applied to the ’PCB pads. This tape serves three primary functions in our assembly process. First, the flexible probe and the chip are secured in place. Second, the 150 µm thick layer of tape creates apertures that are used to hold the solder paste. Third, the tapes act as a protective buffer, mitigating the risk of damage from scratches between the PCB, flexible probe, and chip during assembly.

The flexible probe, prepared on the underside of a polydimethylsiloxane (PDMS) sheet, was then affixed to a slide. This slide, capable of moving in three dimensions, is part of a specialized setup detailed in the supporting materials (Supplementary Fig. 7). Through careful microscopic alignment, we aligned the bonding pads and vias of the probe with the corresponding pads on the PCB and attached the probe to the tape.

Subsequently, another piece of double-sided polyimide tape was attached to the flexible probe, and a scalpel blade was used to fill the apertures created by the tape with the solder paste. The chip was carefully positioned and aligned with the corresponding bonding pads on the flexible probe. Reflow heating is then used to complete the bonding process, forming a fully integrated FLID system.

### Mouse craniotomy and flexible probe implantation

The mechanical strength of the flexible probe is not sufficient to support direct penetration into the cerebral cortex. Before implantation, we employed a tungsten wire as a shuttle device^51^. Specifically, a tip-sharpened tungsten wire with a diameter of 50 µm was attached on the back side of the FLID with polyethylene glycol (PEG m.w. 800) (Supplementary Fig. 10a). The released flexible probe, which has a hole at one end in our design, was temporarily placed on the tungsten wire tip (Supplementary Fig. 10b) and fixed with a diluted PEG solution (m.w. 35k, wt% 10%). After drying under ambient conditions for approximately 1 min, the assembled FLID was ready for implantation.

Before surgery, the mice were anesthetized via isoflurane inhalation using a gas anesthesia machine and received subcutaneous injections of local anesthetics and anti-inflammatory drugs. The mice were fixed on a three-dimensional locator, the head hair was shaved at the surgical site, and the scalp was disinfected with iodophor to reduce discomfort. Erythromycin eye ointment was applied to the eyes of the mice with medical cotton swabs to prevent dry bathing and blindness. During the operation, the scalp was cut off with surgical scissors, the fat and periosteum were removed with medical cotton swabs, the implant position was marked with a locator (hippocampus, AP −1.8 mm, ML −1.8 mm, DV 2 mm), and then the craniotomy was performed with a bone drill. To implant the flexible probe, a tungsten wire was inserted into the target brain tissue together with the flexible probe. Then, the tungsten wire was retracted while the flexible probe was left embedded in tissue, and a steel wire was implanted as a reference electrode. Finally, the device and wire are fixed to the skull using dental cement.

### Behavior recording and analysis

The mice were placed in a 30 × 30 cm cage made of transparent acrylic plates at the same time every day after surgery to allow them to move freely. A high-definition camera was installed in the center above the cage to record the movement trajectory of the mice for behavioral analysis. The trajectory of the mice was visualized by MATLAB, and two-way analysis of variance (ANOVA) was used to analyze the data.

### Tensile testing of FLIDs

We utilized a uniaxial microforce tester (CellScale UniVert S2) to assess the mechanical characteristics of the flexible probe. The force sensor employed had a scale of 4.5 N with a resolution of 0.009 N. Initially, one end of the shank of the flexible probe was secured to the base using a clamp, while the other end was affixed to the sensor with another clamp. Subsequently, the initial length of the shank, set at 5 mm, underwent tensile testing at a speed of 0.125 mm/s. The force sensor monitored the force while the probe was stretched at a constant speed until complete fracture occurred.

### Recycling of the chip

To recycle the Intan chip, the FLID is initially carefully detached from the mouse’s skull by precision milling to remove the dental cement bonding the PCB to the skull. Following FLID removal, we used a heat gun to loosen the chip from the FLID, which was then delicately extracted using tweezers after the solder was melted. To ensure the chip’s continued functionality, it underwent a thorough cleaning process. This involves using a precision electronics cleaning agent (B-2331) to remove any residual oxide layers, solder, or contaminants that may have accumulated on the chip’s surface during the initial bonding. To further confirm that the chip was still functional, we performed two tests. First, the chip was rebonded to a PCB board, following the circuit design of the Intan RHS 16-channel headstage. This home-built headstage was then tested for compatibility with the Intan controller box (128ch RHS stim/recording controller). Second, we performed a sweep frequency response analysis (SFRA) using a conventional KD-MEA rigid probe connected to the headstage. In the test, the probe received a sweep wave from 0–3000 Hz generated from the signal generator in PBS solution (as shown in Supplementary Fig. 9a), and the raw data were obtained from the headstage. The raw data obtained from the headstage are then plotted using software in MATLAB to confirm consistency with the input signal (as demonstrated in Supplementary Fig. 9b).

### Brain section staining

Mouse brain tissue was fixed with 10% formalin and embedded in paraffin. Brain tissue samples were cut into slices, deparaffinized with xylene, rehydrated with low-concentration ethanol, and washed with PBS 3 times for 10 min each. The brain tissue sections were gently shaken with 0.01 M citric acid buffer (pH 7.4) for antigen extraction. After blocking with 10% rabbit serum albumin, the sections were incubated with primary antibody at 4 °C overnight and then incubated with secondary antibody at room temperature for 50 min. The fluorescence signal was observed under a fluorescence microscope.

### Data collection and data analysis

iEEG signals were captured by a custom FPGA device and Verilog code from an ADC chip connected to the electrodes. Data analysis and plot generation were performed using software in MATLAB and custom Python code with Python 3.11.5, SciPy 1.11.3, Numpy 1.24.3, Pandas 2.1.1, and Matplotlib 3.7.2. Involved sorting spikes were sorted by custom parameters in Mountainsort 5 and filtered between 300 Hz and 6000 Hz. Seizure signals in the article are processed with low-pass filtering at 300 Hz and high-pass filtering at 1 Hz. For spectral analysis, an STFT with 0.5 s bins and 50% overlap was used. The corresponding spectrograms illustrate the time-frequency characteristics of each signal. The frequency spectra were calculated using Welch’s method.

### Statistics and reproducibility

Sample sizes were not predetermined using statistical methods. The experimental groups were randomly assigned, and the data are presented as the mean ± s.e.m. or as individual plots. When a normal distribution was assumed, no formal tests were conducted. Two-tailed Student’s *t* tests were used to determine the statistical significance of differences between two groups. Unless stated otherwise, each experiment was independently repeated three times, yielding similar results.

### Animals

All experiments used adult C57BL/6 mice (24 g ~ 30 g at the start of the experiments) provided by the Shanghai Laboratory Animal Research Center.

### Supplementary information

FLID-Supporting materials

## Data Availability

The data that support the findings of this study are available from the corresponding author upon reasonable request.
