# Supplementary material for: Through-polymer, via technology-enabled, flexible, lightweight, and integrated devices for implantable neural probes
Source: Microsyst Nanoeng. 2024 Apr 22;10:54. doi: 10.1038/s41378-024-00691-8 (PMC11035623; doi:10.1038/s41378-024-00691-8)
Supplement: Supplementary file 1 — FLID-Supporting materials [file 41378_2024_691_MOESM1_ESM.docx]

**Supporting materials：**

**Through-Polymer Via Technology-Enabled Flexible, Lightweight, and Integrated Device for Implantable Neural Probes**

*Cunkai Zhou^1,2+^, Ye Tian^2,3,+^, Gen Li^2,3,+^, YiFei Ye^2^, Lusha Gao^2^, Jiazhi Li^2^, Ziwei Liu^2^, Haoyang Su^3,4^, Yunxiao Lu^1,2^, Meng Li^3,4^, Zhitao Zhou^3,4^, Xiaoling Wei^3,4^, Lunming Qin^1^*, Tiger H. Tao^,2,3,4,5,6,7,8,9,10,^*, Liuyang Sun^1,2,3,4^**

^1^College of Electronics and Information Engineering, Shanghai University of Electric Power, Shanghai, China

^2^2020 X-Lab, Shanghai Institute of Microsystem and Information Technology, Chinese Academy of Sciences, Shanghai, China

^3^School of Graduate Study, University of Chinese Academy of Sciences, Beijing, China

^4^State Key Laboratory of Transducer Technology, Shanghai Institute of Microsystem and Information Technology, Chinese Academy of Sciences, Shanghai, China

^5^Center of Materials Science and Optoelectronics Engineering, University of Chinese Academy of Sciences, Beijing, China

^6^School of Physical Science and Technology, ShanghaiTech University, Shanghai, China

^7^Center for Excellence in Brain Science and Intelligence Technology, Chinese Academy of Sciences, Shanghai, China

^8^Neuroxess Co., Ltd. (Jiangxi), Nanchang, Jiangxi, China

^9^Guangdong Institute of Intelligence Science and Technology, Hengqin, Zhuhai, Guangdong, China

^10^Tianqiao and Chrissy Chen Institute for Translational Research, Shanghai, China

+These authors contributed equally.

*Corresponding author:

Email address: lunming.qin@shiep.edu.cn (Lunming Qin); [tiger@mail.sim.ac.cn](mailto:tiger@mail.sim.ac.cn) (Tiger H. Tao); [liuyang.sun@mail.sim.ac.cn](mailto:liuyang.sun@mail.sim.ac.cn) (Liuyang Sun).


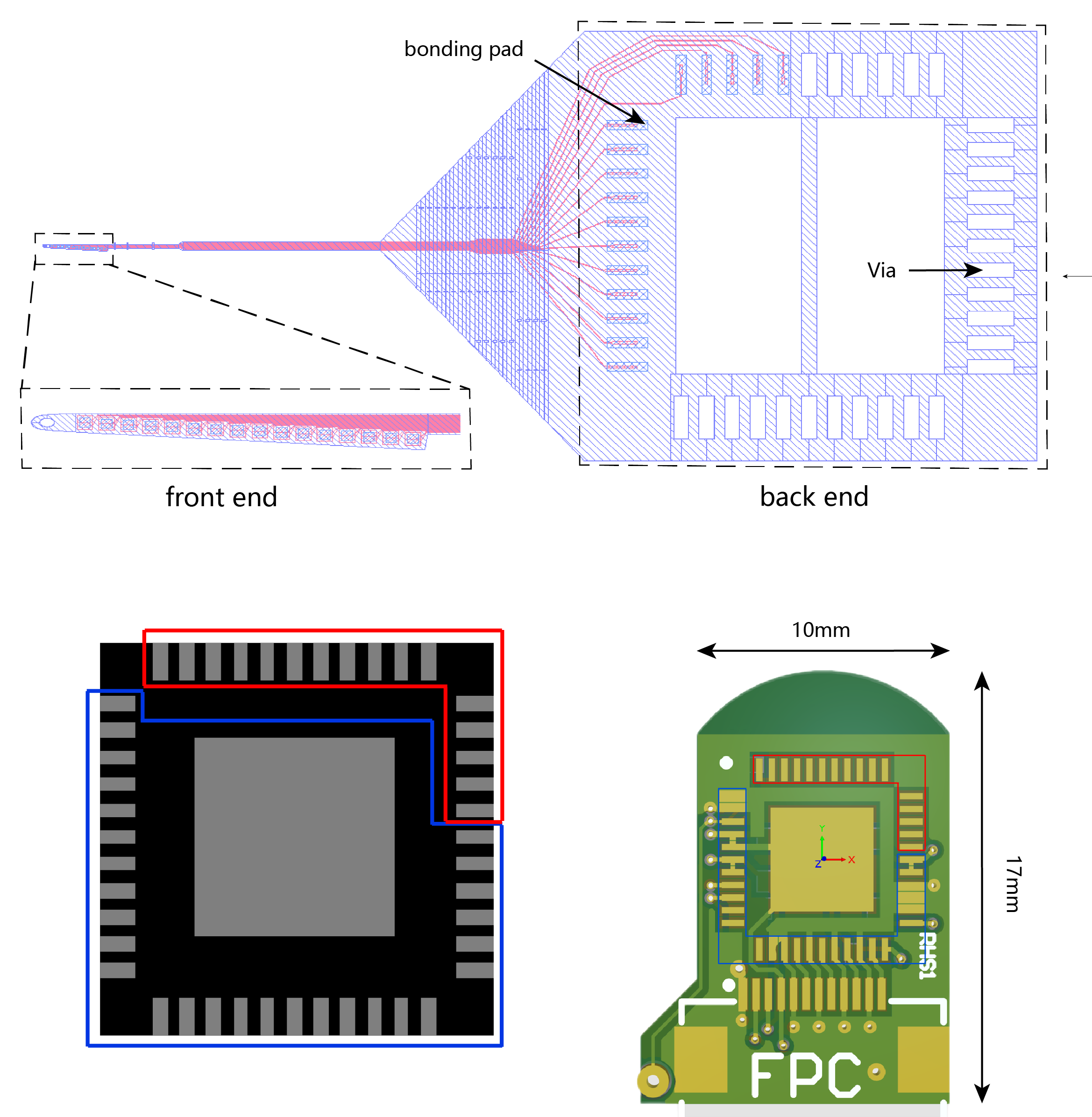


**SFig. 1** Intan chip bottom schematic**.** A total of 45 pins, red part 16 pins allocated for neural signal acquisition and blue part 29 pins dedicated to communicating with the host computer software.


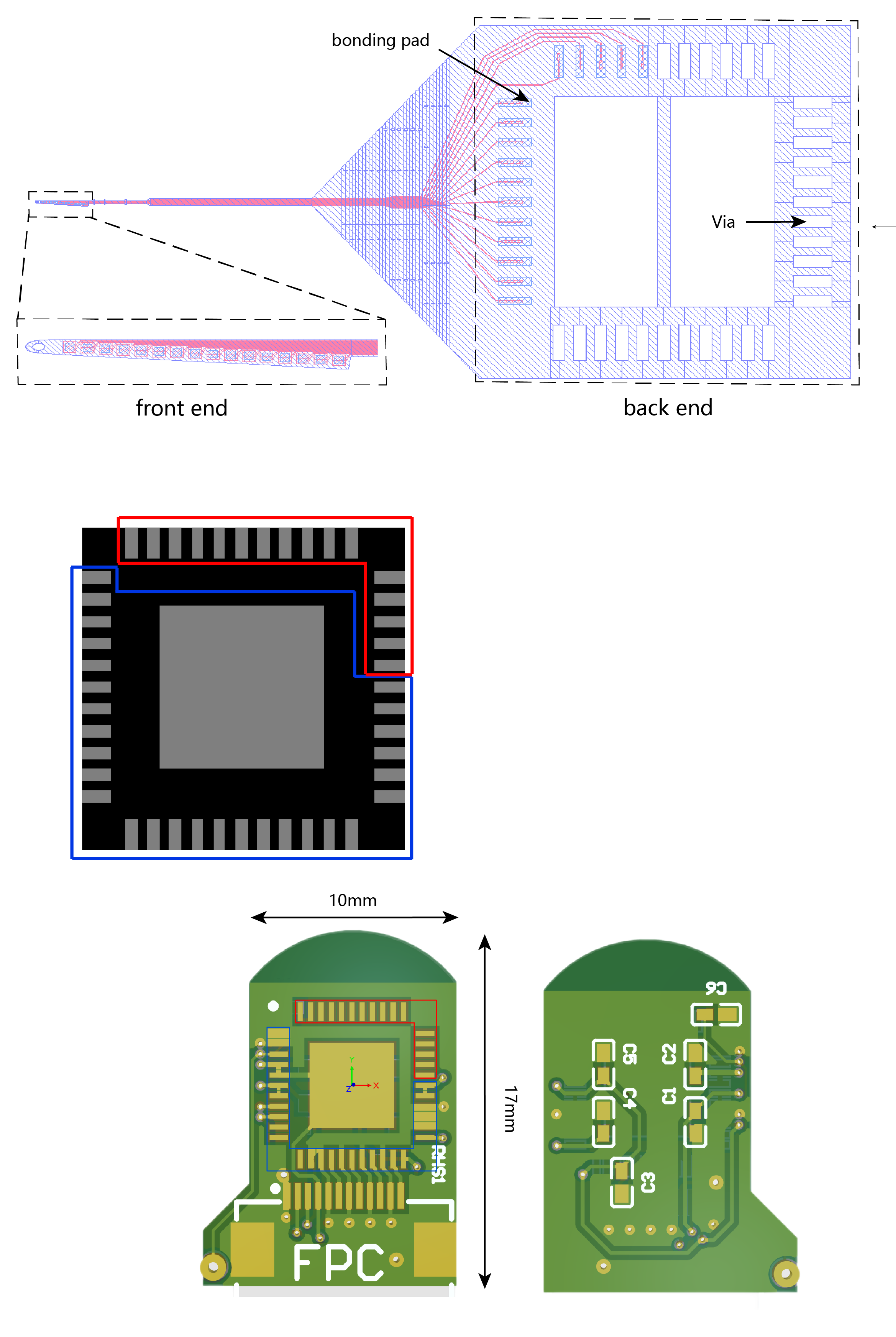


**SFig. 2** Overall schematic diagram of flexible probe.


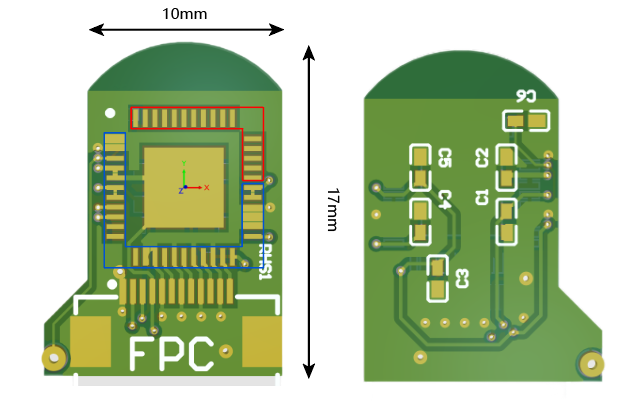


**SFig. 3** Schematic diagram of the PCB substrate.


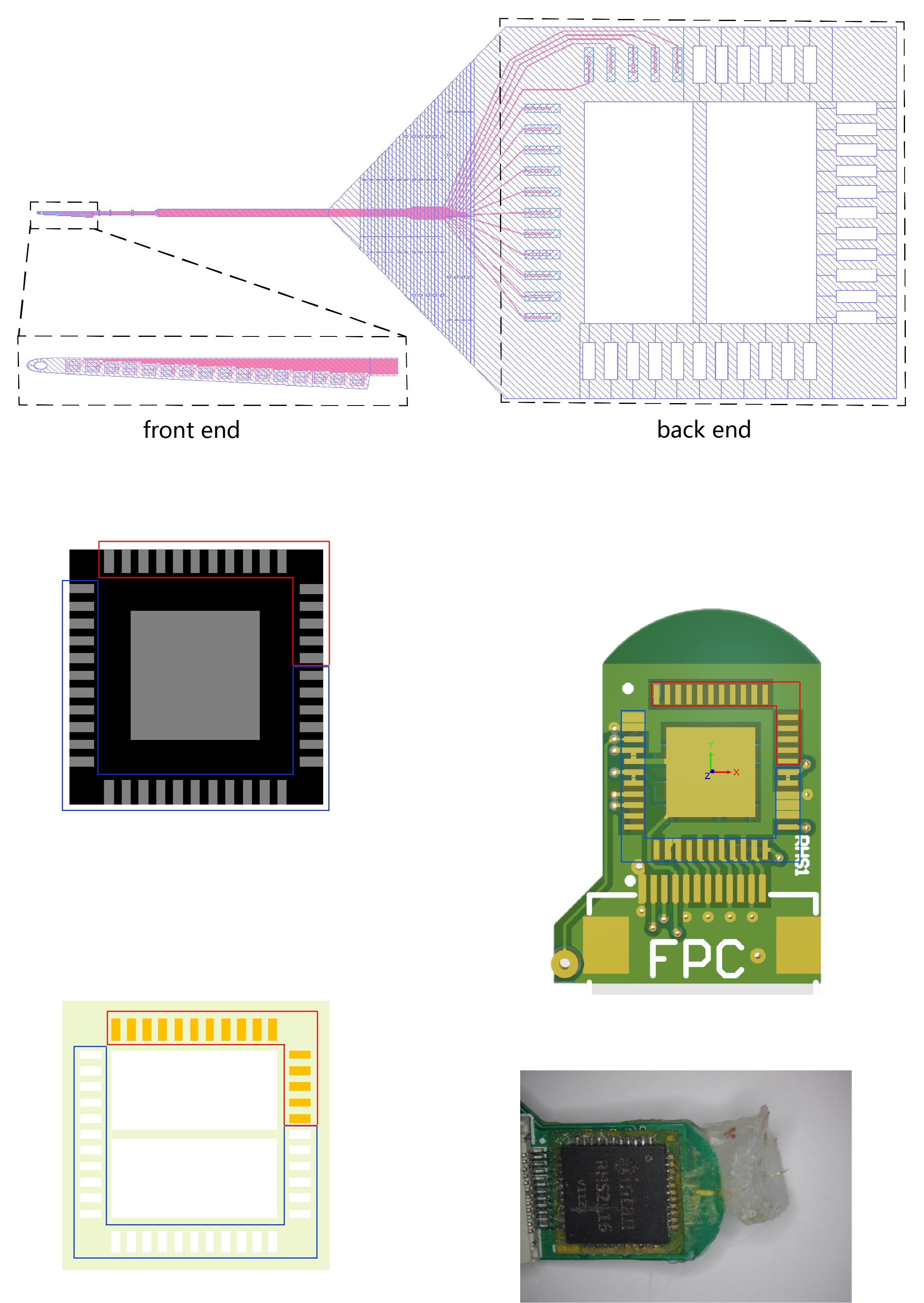


**SFig. 4** Schematic diagram of the complete device removal.


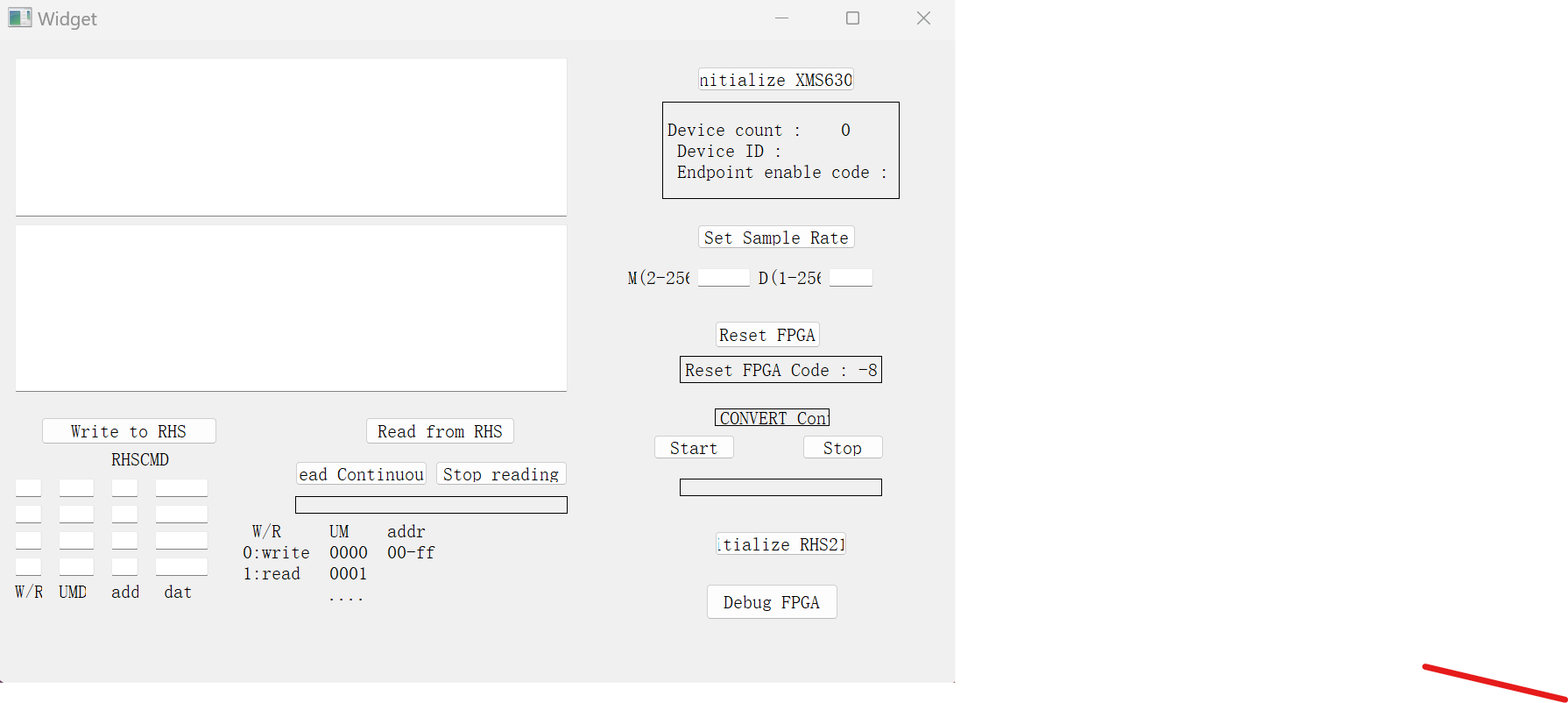


**SFig. 5** Host computer software operation interface.


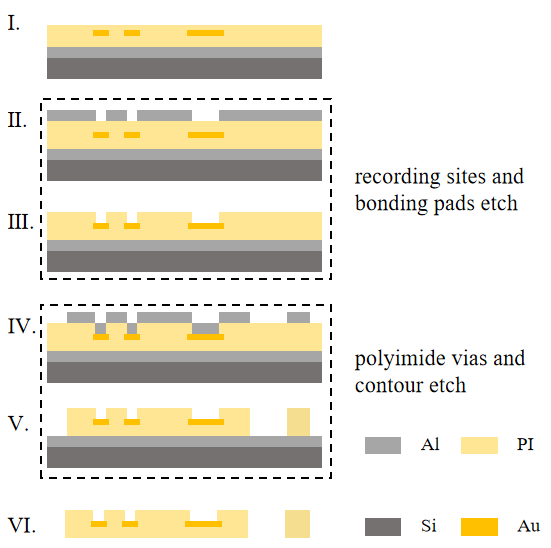


**SFig. 6 Schematic diagram of polyimide etching.** I) Before the polyimide etching. Ⅱ) The Al mask patterned for recording sites and bonding pads. Ⅲ) The top PI etched. Ⅳ) The Al mask patterned for contour and polyimide vias. V) The dual layers of PI etched. Ⅵ) The flexible probe released.


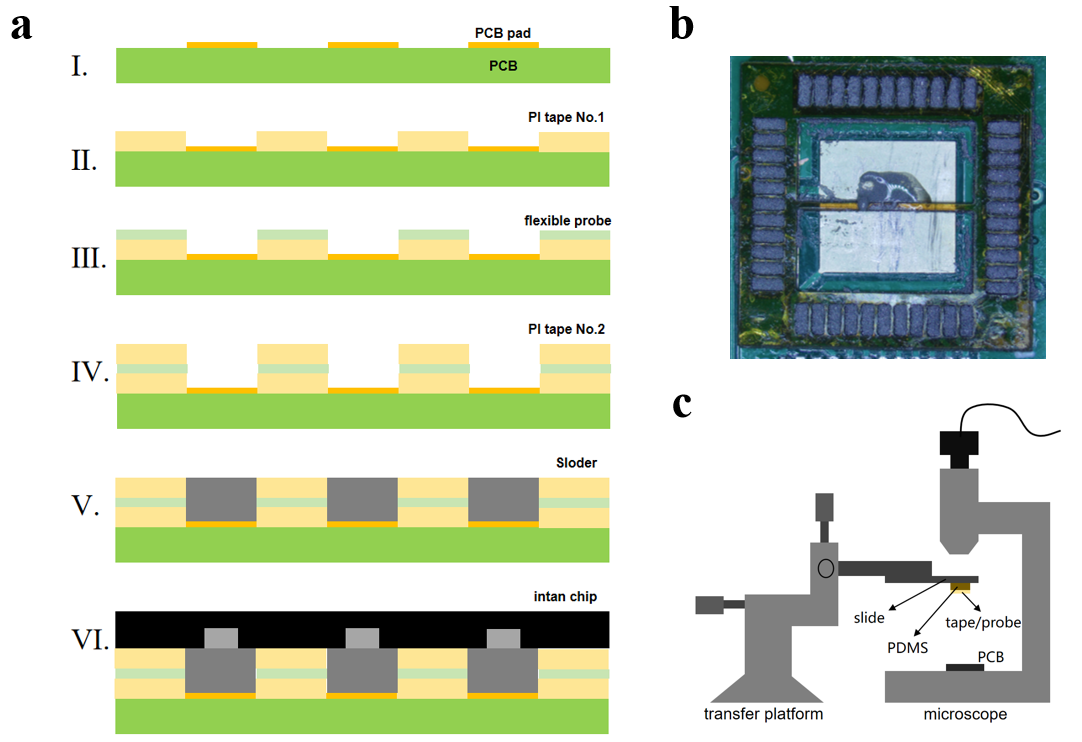


**SFig. 7 The use of double-sided polyimide tapes in the TPV method.** **a,** Flow chart of the use of the polymide tape. **b,** The solder was filled in the polyimide vias. **c,** Using the vertical stacking manipulator to bond FLID, the PDMS is employed to transfer either the tape or the probe.


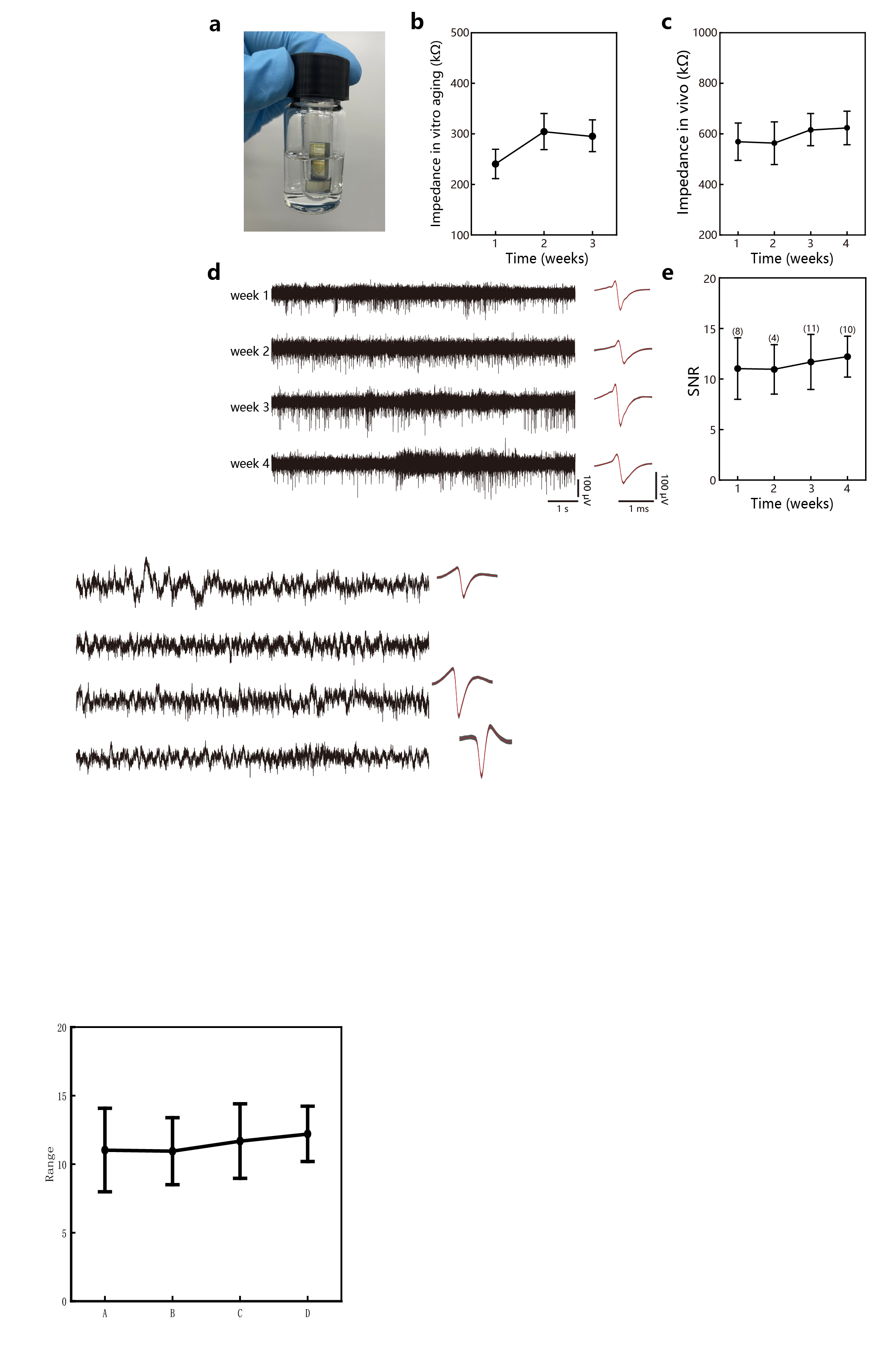


**SFig. 8 Stability analysis of the Pt-Ir flexible probe. a,** The Pt-Ir flexible probe was immersed in PBS for aging testing to simulate physiological conditions. **b,** Impedance change of the Pt-Ir flexible probes at 60 ℃ (n = 56 recording sites of 5 probes). **c,** Impedance change of the Pt-Ir flexible probe in one mouse for 4 weeks (n = 24 recording sites of 3 mice). **d,** One channel EEG signal recorded over 4 weeks. Left: 10s real-time recording trace. Right: the same neuron spike isolated from the recording trace and plotted in red, and gray represents noise. **e,** SNR changes of the Pt-Ir flexible probe in vivo over 4 weeks, the number in bracket above bar represent the number neurons sorted out during the recording session.


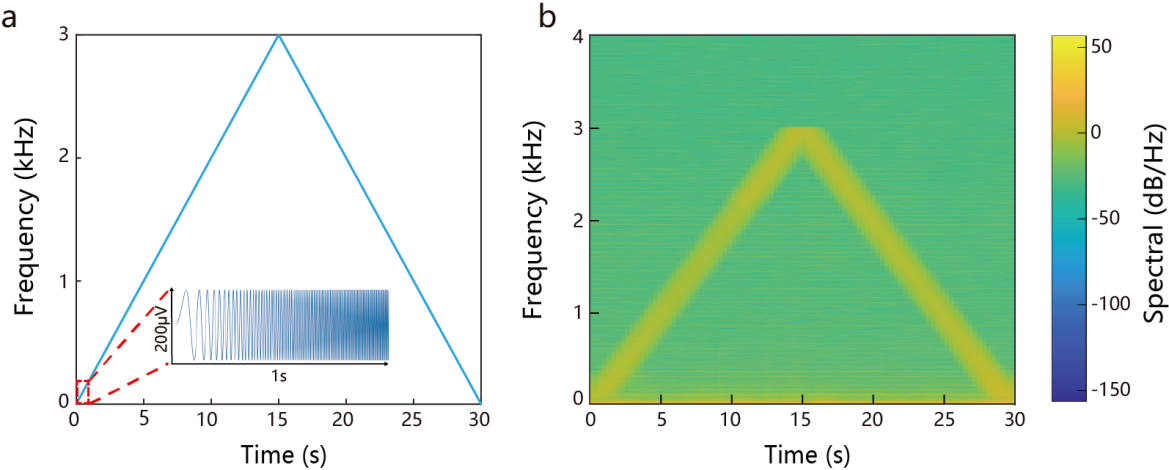


**SFig. 9 Sweep Frequency Response Analysis test. a,** The waveform generated by the signal generator. **b,** The reduced waveform after chip processing.


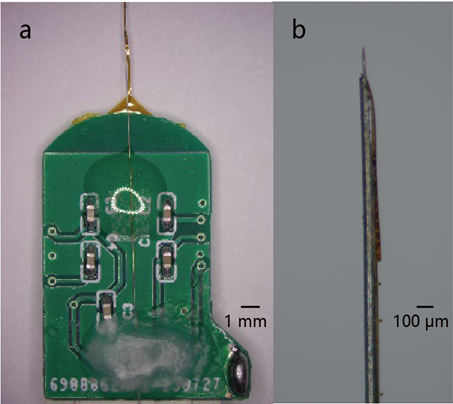


**SFig. 10** **Photograph of the tungsten wire assembly before FLID implantation.** **a,** Tungsten wire was fixed on the back of FLID using PEG. **b,** The hole at the front end of the flexible probe is sleeved on the tungsten wire, and fixed with PEG.


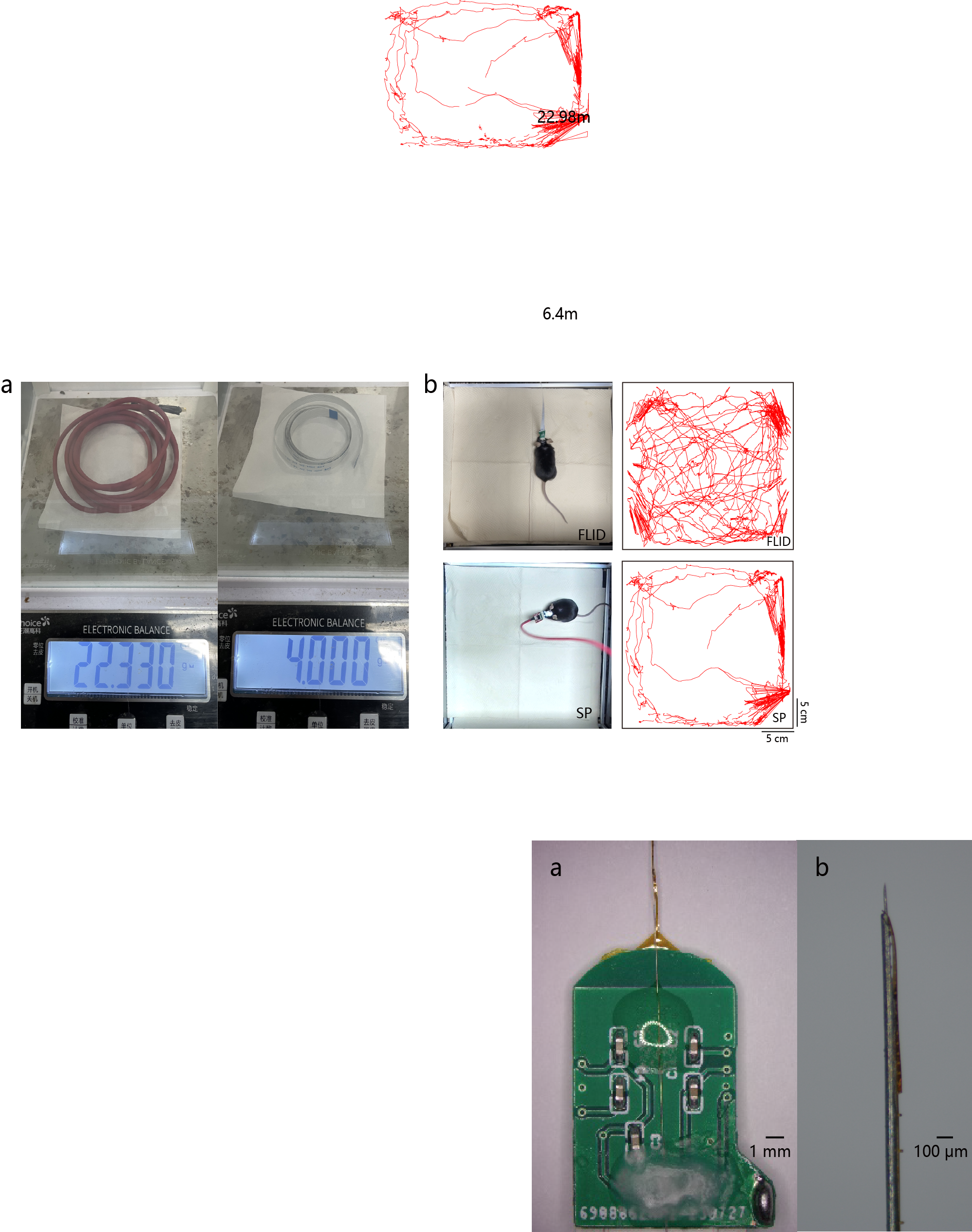


**SFig. 11** **Weight of two cables and open-field tests with the cables connected**. **a,** The SPI interface cable and the JS05B-12P cable are each 1.8 m long, with weights of 22.33 g and 4 g, respectively. **b,** Travel traces of FLID mice and SP mice in a 10 min recording session.
